# Low-level laser irradiation promotes proliferation of cryopreserved adipose-derived stem cells

**DOI:** 10.1590/S1679-45082017AO3991

**Published:** 2017

**Authors:** Fernanda Ginani, Diego Moura Soares, Hugo Alexandre de Oliveira Rocha, Carlos Augusto Galvão Barboza

**Affiliations:** 1 Universidade Federal do Rio Grande do Norte, Natal, RN, Brazil.; 2 Universidade Federal de Pernambuco, Recife, PE, Brazil.

**Keywords:** Adipose tissue, Cryopreservation, Laser therapy, Stem cells, Cell proliferation, Tecido adiposo, Criopreservação, Terapia a laser, Células--tronco, Proliferação celular

## Abstract

**Objective:**

To evaluate the effect of low-level laser irradiation on proliferation and viability of murine adipose-derived stem cells previously submitted to cryopreservation.

**Methods:**

Adipose-derived stem cells were isolated from inguinal fat pads of three mice, submitted to cryopreservation in fetal bovine serum with 10% dimethylsulfoxide for 30 days and then thawed and maintained in normal culture conditions. Culture cells were either irradiated or not (control) with an InGaAIP diode laser at zero and 48 hours, using two different energy densities (0.5 and 1.0J/cm^2^). Cell proliferation was evaluated by trypan blue exclusion method and MTT assay at intervals of zero, 24, 48, and 72 hours after the first laser application. Cell viability and apoptosis of previously cryopreserved cells submitted to laser therapy were evaluated by flow cytometry.

**Results:**

The Irradiated Groups (0.5 and 1.0J/cm^2^) showed an increased cell proliferation (p<0.05) when compared to the Control Group, however no significant difference between the two energy densities was observed. Flow cytometry revealed a percentage of viable cells higher than 99% in all groups.

**Conclusion:**

Low-level laser irradiation has stimulatory effects on the proliferation of adipose-derived stem cells previously submitted to cryopreservation.

## RESUMO

**Objetivo:**

Avaliar o efeito do *laser* de baixa intensidade na proliferação e na viabilidade de células-tronco derivadas de tecido adiposo murinas previamente submetidas à criopreservação.

**Métodos:**

Células-tronco derivadas de tecido adiposo foram isoladas da região inguinal de três camundongos, submetidas à criopreservação em soro fetal bovino com 10% de dimetilsulfóxido por 30 dias e, depois, descongeladas e mantidas em condições normais de cultivo. As células cultivadas foram irradiadas ou não (controle) com um *laser* de diodo InGaAIP nos intervalos de zero e 48 horas, utilizando duas densidades de energia diferentes (0,5 e 1,0J/cm^2^). A proliferação celular foi avaliada pelo método de exclusão de azul de tripan e ensaio MTT, nos intervalos de zero, 24, 48 e 72 horas após a primeira aplicação do *laser*. A viabilidade celular e a apoptose das células previamente criopreservadas submetidas à laserterapia foram avaliadas por citometria de fluxo.

**Resultados:**

Os Grupos Irradiados (0,5 e 1,0J/cm^2^) apresentaram aumento da proliferação celular (p<0,05) quando comparados ao Grupos Controle, porém não foi observada diferença significativa entre as duas densidades de energia. A citometria de fluxo revelou percentagem de células viáveis superior a 99% em todos os grupos.

**Conclusão:**

O *laser* de baixa intensidade tem efeitos estimuladores sobre a proliferação de células-tronco derivadas de tecido adiposo previamente submetidas à criopreservação.

## INTRODUCTION

Adipose-derived stem cells (ADSC) have been widely investigated because they are readily accessible from subcutaneous liposuction, have few ethical considerations, and present multiple differentiation potential. These characteristics make them a viable option in regenerative medicine.^(^ ^1^ ^)^

With the development of cellular therapy and tissue engineering, a considerable number of cells is required. In this regard, several studies have evaluated the effect of low-level laser irradiation (LLLI) on the proliferation of mesenchymal stem cells (MSC) obtained from different sources,^(^ ^2^ ^)^ and satisfactory results were reported on ADSC.^(^ ^3^ ^-^ ^7^ ^)^ Thus, laser therapy has emerged as an alternative to stimulate *in vitro* cell proliferation.

Another possibility for MSC collection is to keep them stored for long periods of time for subsequent clinical application, with no loss of their function, thereby requiring cryopreservation. The purpose of cryopreservation is to reversibly cease all biological functions of living tissues at temperatures ranging from -80 to -196°C. This technique has been used for several decades in MSC obtained from different sources, such as bone marrow,^(^ ^8^ ^)^ adipose tissue,^(^ ^9^ ^,^ ^10^ ^)^ umbilical cord,^(^ ^11^ ^)^ dental pulp^(^ ^12^ ^,^ ^13^ ^)^ and periodontal ligament.^(^ ^14^ ^)^

## OBJECTIVE

To evaluate the effect of low-level laser irradiation on proliferation and viability of primary culture of murine adipose-derived stem cells, which were previously submitted to cryopreservation.

## METHODS

### Cell isolation and culture

This study was approved by the Animal Research Ethics Committee of the *Universidade Federal do Rio Grande do Norte*, Brazil (protocol 036/2012), and followed the guiding principles of the Declaration of Helsinki. The experiments were performed at the Departments of Morphology and Biochemistry of the organization, from 2013 to 2014. Fragments of adipose tissue were isolated from inguinal fat pads of three 2-month old male Swiss albino mice, according to a previously described protocol.^(^ ^9^ ^)^ Briefly, the fragments were washed with minimum essential medium (α-MEM; Cultilab, Brazil), supplemented with antibiotics and antifungal agents (Gibco, United States) and then submitted to enzyme digestion with a solution containing 3mg/mL collagenase I (Gibco, United States) for 1 hour, at 37°C. Cells were then cultured in α-MEM supplemented with 10% fetal bovine serum (FBS; Gibco, United States), and maintained at 37°C, in 5% carbon dioxide until reaching a confluence between 70 and 90%.

The multipotential nature of the cells was confirmed on second passage (P2), after cryopreservation and thawing, by expression of CD44 and CD29 (BD Biosciences, United States) in more than 98% of cells. Furthermore, after culturing the cells in osteogenic and adipogenic differentiation medium (StemPro^®^ Differentiation Kits, Invitrogen Corp., United States) for 21 days, the cells presented the characteristic morphology of osteoblastic and adipose cells when observed under a light microscope.

### Cryopreservation

In the first passage (P1), the cells were submitted to cryopreservation in FBS with 10% dimethylsulfoxide with gradual decrease of temperature (2 hours at 4°C, 18 hours at -20°C, and then stored at -80°C). These cells were cryopreserved for 30 days and then thawed and maintained in normal culture conditions.

### Laser irradiation

In the third passage (P3), cells were divided into three groups according to treatment: (1) control: no irradiation; (2) 0.5J/cm^2^: cells irradiated with a dose of 0.5J/cm^2^; (3) 1.0J/cm^2^: cells irradiated with a dose of 1.0J/cm^2^. Irradiations were performed at zero and 48 hours with an InGaAlP diode laser (Kondortech Bio Wave LLLT Dual, Brazil) following the parameters listed in chart 1. The cells were plated in such a way that wells were left empty between the seeded wells in order to prevent the unintentional dispersion of light during laser irradiation.

**Chart 1:** Laser parameters

Parameters	Reference
Power	30mW
Wavelength	660nm
Mode of action	Continuous
Output area	0.03cm^2^
Tip diameter	0.01cm^2^
Energy density	0.5 and 1.0J/cm^2^
Irradiation time	16 seconds (0.5J/cm^2^) and 33 seconds (1.0J/cm^2^)
Application mode	Probe was directed perpendicular to each plate at a distance of 0.5cm from the cells

### Analysis of the effect of laser treatment on cell proliferation

The analyzes of cell proliferation were performed at intervals of zero, 24, 48 and 72 hours, after the first laser application in the Control Group (non-irradiated) and Irradiated Groups by trypan blue exclusion method and 3-(4,5-dimethylthiazol-2-yl)-2,5-diphenyltetrazolium bromide (MTT) tetrazolium reduction assay. For trypan blue staining, cells were cultured in 24-well plates, at a density of 3x10^4^ cells/well. Cell counting was carried out on four wells/group at each time interval by two blinded and previous calibrated examiners using a Neubauer chamber. For MTT assay, cells were cultured in 96-well plates at a density of 5x10^3^ cells/well with four wells for each group (control, 0.5J/cm^2^ and 1.0J/cm^2^). Cells were incubated in 100µL culture medium with 1mg/mL of MTT for 4 hours and then the colorimetric product (formazan) was solubilized with 100µL of dimethylsulfoxide. The absorbance of the samples was monitored in an ELISA reader at 570nm.

### Analysis of cell viability

Cell viability and apoptosis of previously cryopreserved cells submitted to laser therapy were evaluated by flow cytometry using the FITC/Annexin V Dead Cell Apoptosis Kit with FITC annexin and propidium iodide (PI; Invitrogen Corp., Carlsbad, CA, USA). For this purpose, the cells were triple cultured in six well plates at a density of 2x10^5^ cells/well. After 72 hours of culture, the cells were trypsinized, collected and washed with ice-cold FBS. The supernatant was discarded and the cells were resuspended in 200µL 1X binding buffer. Next, 3µL annexin V-FITC and 1µL 100µg/mL PI were added. The cells were incubated for 15 minutes at room temperature protected from light. After incubation, 400µL 1X binding buffer for annexin V was added, and the cells were analyzed in a flow cytometer, measuring the fluorescence emitted at 530 and 575nm.

### Statistical analysis

Differences between groups at each time point were analyzed by the Kruskal-Wallis and Mann-Whitney tests, considering a level of significance of 5% (p<0.05).

## RESULTS

**Figure 1:**
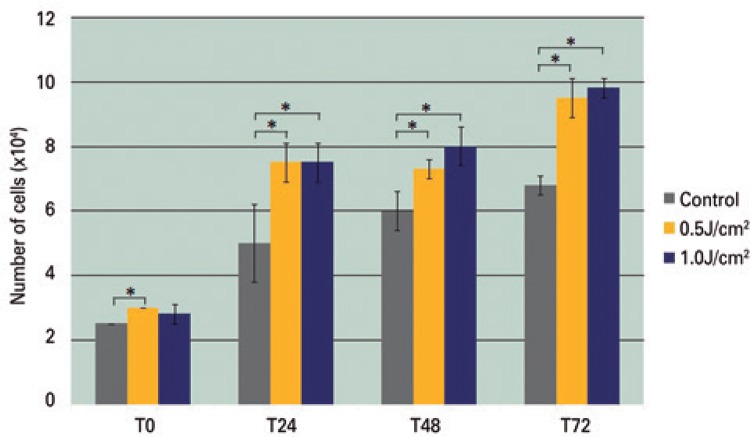
Number of adipose-derived stem cells at the different time points studied. Data are presented in mean±standard deviation from four wells/group

The means of ADSC analyzed by the trypan blue exclusion method in the different groups are showed in figure 1. The Irradiated Groups (0.5 and 1.0J/cm^2^) showed an increased cell proliferation (p<0.05) when compared to the Control Group at intervals of 24, 48 and 72 hours (Figure 1), however no significant difference between the two energy densities was observed.

* p<0.05, Mann-Whitney test.

**Figure 2:**
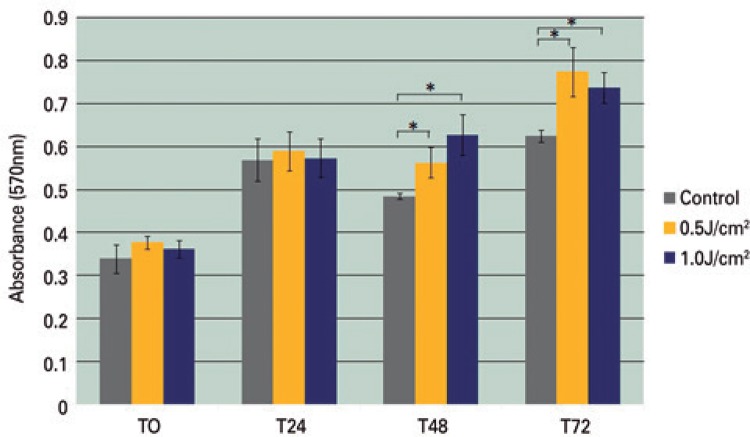
Mitochondrial activity of adipose-derived stem cells measured by MTT assay. Data are presented in mean±standard deviation of absorbance from four wells/group

The pattern of mitochondrial activity analyzed by the MTT assay showed similar results to cell counting by Trypan blue assay, with the Irradiated Groups showing a significantly higher activity of MTT at intervals of 48 and 72 h (p<0.05) when compared to the Control Group (Figure 2).

* p<0.05, Mann-Whitney test.

**Figure 3:**
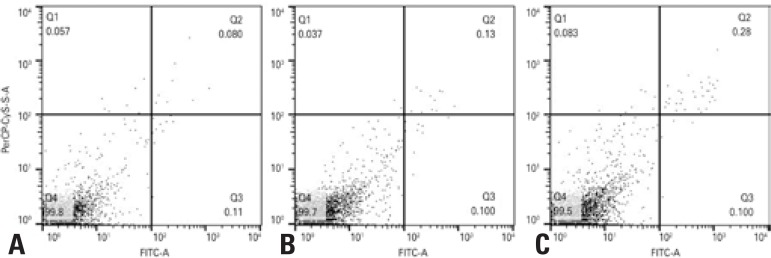
Immunostaining of adipose-derived stem cells previously submitted to cryopreservation with annexin V/PI. (A) Control, (B) irradiated with 0.5J/cm2 and (C) irradiated with 1.0J/cm2

Q1: annexin V negative/PI positive; Q2: annexin V positive/PI positive; Q3: annexin V positive/PI negative; Q4: annexin V negative/PI negative.

Flow cytometry revealed a percentage of viable cells higher than 99% in all groups (Figure 3), indicating that viability of the cells previously cryopreserved was not affected by the laser therapy throughout the experiment.

## DISCUSSION

Considering the increasing number of liposuction procedures in recent years facilitating attainment of adipose tissue, cryopreservation of this tissue for subsequent clinical use has been widely studied.^(^ ^15^ ^-^ ^17^ ^)^ With the purpose of storing organs and cells for long periods of time, different cryopreservation protocols have been tested. In the present study, ADSC were cryopreserved for a period of 30 days, with maintenance of their viability after thawing, and with results in accordance with other studies.^(^ ^9^ ^,^ ^15^ ^,^ ^17^ ^,^ ^18^ ^)^

In this study, we chose to cryopreserve the cells at -80°C because it is a simpler, accessible and low-cost technique. Previous studies evaluated the cryopreservation of dental pulp stem cells for 6 months^(^ ^13^ ^,^ ^19^ ^)^ and periodontal ligament stem cells for 30 days,^(^ ^14^ ^)^ and concluded that cryopreservation at minus 80°C exhibits similar cell viability rates compared with cells cryopreserved in liquid nitrogen.

One of the difficulties encountered in the cryopreservation process is the restoration of the biological cell properties after thawing. Usually the initial cell yield is low, and thus LLLI can be an auxiliary tool to promote biostimulation of diverse cell types. This work is the first to demonstrate the positive effect of LLLI on proliferation of MSC previously submitted to cryopreservation, confirming the increased proliferative rate previously demonstrated in fresh (not cryopreserved) MSC obtained from various sources, such as bone marrow,^(^ ^20^ ^)^ adipose tissue,^(^ ^4^ ^)^ periodontal ligament,^(^ ^21^ ^)^ and dental pulp.^(^ ^22^ ^,^ ^23^ ^)^ The exception is one study that verified a higher number of colony-forming units (CFU) in cryopreserved peripheral blood progenitor cells exposed to a single irradiation of 1.0J/cm^2^.^(^ ^24^ ^)^

When aiming for cell biostimulation, the wavelength and energy density are parameters that must be considered. The literature reports that the spectrum of visible light (600 to 700nm) provides more effective results for *in vitro* cell biostimulation, being the most used in the LLLI studies on stem cells.^(^ ^2^ ^)^ In relation to the energy density, it is known that the biostimulation process can be achieved with very low doses such as 0.001J/cm^2^ to larger doses of 10J/cm^2^.^(^ ^25^ ^)^ In the present study and using a wavelength of 660nm, it was found that both 0.5 and 1.0J/cm^2^ were effective in promoting increased proliferation of cryopreserved ADSC when compared with a non-irradiated group, corroborating previous findings in fresh ADSC irradiated with the same energy densities (0.5 and 1.0J/cm^2^).^(^ ^8^ ^)^ Other studies showed the biostimulating effect of LLLI in fresh ADSC using a wavelength of 660nm and a single dose of 5J/cm^2^.^(^ ^3^ ^-^ ^5^ ^)^ The only study assessing the effect of LLLI with wavelengths higher than 800nm on ADSC showed a 830nm laser (0.05J/cm^2^) promoted an increase in cell proliferation of ADSCs compared to the Control Group.^(^ ^7^ ^)^

## CONCLUSION

Low-level laser irradiation has stimulatory effects on the proliferation of murine adipose-derived stem cells previously submitted to cryopreservation. Thus, the association of cryopreservation and laser therapy may represent an important advance in the technical procedures of cell therapy and tissue engineering.
